# Enhancing Adult Asthma Management: A Review on the Utility of Remote Home Spirometry and Mobile Applications

**DOI:** 10.3390/jpm14080852

**Published:** 2024-08-11

**Authors:** Norbert Wellmann, Monica Steluta Marc, Emil Robert Stoicescu, Camelia Corina Pescaru, Ana Adriana Trusculescu, Flavia Gabriela Martis, Ioana Ciortea, Alexandru Florian Crisan, Madalina Alexandra Balica, Diana Raluca Velescu, Ovidiu Fira-Mladinescu

**Affiliations:** 1 Center for Research and Innovation in Personalised Medicine of Respiratory Diseases (CRIPMRD), “Victor Babes” University of Medicine and Pharmacy Timisoara, Eftimie Murgu Square 2, 300041 Timisoara, Romania; norbert.wellmann@umft.ro (N.W.); pescaru.camelia@umft.ro (C.C.P.); ana.trusculescu@umft.ro (A.A.T.); flavia.martis@umft.ro (F.G.M.); crisan@umft.ro (A.F.C.); velescu.diana@umft.ro (D.R.V.); mladinescu@umft.ro (O.F.-M.); 2 Pulmonology University Clinic, “Victor Babes” University of Medicine and Pharmacy Timisoara, Eftimie Murgu Square 2, 300041 Timisoara, Romania; 3 Doctoral School, “Victor Babes” University of Medicine and Pharmacy Timisoara, Eftimie Murgu Square 2, 300041 Timisoara, Romania; stoicescu.emil@umft.ro (E.R.S.); ioana.ciortea@umft.ro (I.C.); madalina.balica@umft.ro (M.A.B.); 4 Research Center for Pharmaco-Toxicological Evaluations, “Victor Babes” University of Medicine and Pharmacy Timisoara, Eftimie Murgu Square No. 2, 300041 Timisoara, Romania; 5 Radiology and Medical Imaging University Clinic, “Victor Babes” University of Medicine and Pharmacy Timisoara, Eftimie Murgu Square No. 2, 300041 Timisoara, Romania; 6 Research Center for the Assessment of Human Motion, Functionality and Disability (CEMFD), “Victor Babes” University of Medicine and Pharmacy Timisoara, Eftimie Murgu Square 2, 300041 Timisoara, Romania; 7 Infectious Diseases University Clinic, Department of Infectious Diseases, “Victor Babes” University of Medicine and Pharmacy Timisoara, Eftimie Murgu Square 2, 300041 Timisoara, Romania

**Keywords:** asthma control, home monitoring systems, telemedicine, portable spirometers, self-management, chronic diseases, digital coaching, asthma management

## Abstract

Asthma is a prevalent chronic disease, contributing significantly to the global burden of disease and economic costs. Despite advances in treatment, inadequate disease management and reliance on reliever medications lead to preventable deaths. Telemedicine, defined as the use of information and communication technology to improve healthcare access, has gained global attention, especially during the COVID-19 pandemic. This systematic review examines the effectiveness of home monitoring systems in managing severe asthma. A systematic literature search was conducted in PubMed, Web of Science, Scopus, and Cochrane Library, focusing on studies from 2014 to 2024. Fourteen studies involving 9093 patients were analyzed. The results indicate that telemedicine, through tools such as mobile applications and portable spirometers, positively impacts asthma control, self-management, and quality of life. Home spirometry, in particular, shows strong agreement with clinic spirometry, offering a feasible alternative for continuous monitoring. Digital coaching and machine learning-based telemedicine applications also demonstrate significant potential in improving asthma outcomes. However, challenges such as technology accessibility, data privacy, and the need for standardized protocols remain. This review highlights the promise of telemedicine in asthma management and calls for further research to optimize its implementation and address existing barriers.

## 1. Introduction

### 1.1. Asthma

According to the 2019 estimation by the Global Burden of Disease collaboration, asthma affects a substantial number of individuals worldwide, with as many as 262 million people impacted by this prevalent chronic disease. It significantly contributes to the burden of disease, leading to premature mortality and a diminished quality of life across various age groups and geographical regions globally. In the global context, asthma ranks 24th among the foremost causes of years lives with a disability and 34th among the principal contributors to the burden of disease, as assessed by disability-adjusted life years [1,2,3].

Asthma’s economic impact remains a significant global concern, evidenced by both direct and indirect costs. Implementing strategies aimed at enhancing access and adherence to evidence-based treatments has proven effective in mitigating the economic burden of asthma, not only in developed nations but also in developing countries. Despite a decline in age-specific asthma mortality rates in several countries over the past decade, approximately 1000 individuals succumb to asthma daily, presenting a grave issue given the preventable nature of many of these deaths. Although these mortality rates have decreased, the overall number of asthma-related fatalities has shown little change due to the aging of the population. Preventable deaths from asthma persist due to inadequate disease management, often attributed to an excessive reliance on reliever medications rather than preventive measures. Addressing this issue is imperative for ensuring a reduction in avoidable asthma-related deaths [1,2,4].

Asthma is a multifaceted and heterogeneous ailment, commonly characterized by sustained airway inflammation caused by a variety of triggers. It is clinically identified through a comprehensive assessment of respiratory manifestations, encompassing whizzing, dyspnea, a feeling of tightness in the chest, and cough, which demonstrate fluctuation over time and in intensity, along with periodic expiratory airflow obstruction. Asthma is commonly linked with airway hyperresponsiveness and airway inflammation, yet these factors alone are neither necessary nor sufficient for diagnostic purposes. Frequently termed asthma phenotypes, identifiable clusters of demographic clinical, and/or pathophysiological characteristics do not exhibit a strong correlation with specific pathological processes or treatment responses. Spirometry is a helpful diagnostic technique for tracking individuals with recognized respiratory disorders as well as for determining the etiology of unexplained respiratory symptoms. For the diagnosis of obstructive airway illnesses, such as asthma and chronic obstructive pulmonary disease (COPD), it is still the gold standard test [1,5,6].

### 1.2. Telemedicine

Telemedicine is a means of improving access to healthcare and is defined as information and communication technology. It can enhance access to healthcare in both urban and rural areas, providing opportunities for remote monitoring and medical education [7,8,9].

There is significant global attention on the capacity of telehealth care to enhance the accessibility, quality, safety, and cost efficiency of healthcare services. In respiratory pathologies, the devices provided by telemedicine for patient home monitoring allow clinicians to monitor the real-time progress of the pathology and adherence to treatment, check inhalation techniques, and offer consultation as needed [10,11].

Before the onset of the COVID-19 pandemic, physicians utilized a hybrid model, combining telemedicine with on-site visits. Throughout the pandemic, telemedicine has been preferred as a care delivery option where the presence of a medical professional was not strictly necessary. This approach minimized contact with healthcare facilities, prioritizing the protection of medical staff and patients alike. Post-pandemic, several healthcare systems, in collaboration with private entities, are considering adopting telemedicine as a standard of care [12,13].

Health mobile applications are primarily designed to monitor activities associated with specific pathologies, providing users with relevant information regarding their overall health and well-being or to simplify the autonomous management of specific drug treatments. These applications are configured to record and analyze relevant medical parameters, such as symptoms specific to a condition, metabolic levels, blood pressure, or cardiac activity. They facilitate the monitoring of the user’s clinical progression, providing useful data for individualized health management. In addition to medical monitoring aspects, these applications aim to educate users on various health-related aspects. They can provide relevant information about dietary regimens and recommended physical exercises or stress management strategies, thus contributing to promoting a healthy lifestyle. Moreover, mobile health applications are designed to facilitate the management of self-pharmacological treatments. They offer functionalities such as periodic notifications for medication administration, proper monitoring of dosage regimens, and highlighting the need for prescription renewal, thereby ensuring patient compliance with the established therapeutic protocol. It is essential to emphasize that the use of these applications should take place under the supervision and guidance of a medical professional to ensure the correct interpretation of data and to ensure that therapeutic interventions are appropriate and effective in the specific context of each patient [14,15].

Telemedicine has emerged as an efficient alternative in delivering pulmonary rehabilitation programs remotely, thereby overcoming accessibility barriers for patients who cannot physically attend respiratory rehabilitation sessions due to other obligations or residing in remote geographic areas. Through the telemonitoring of bioparameters, proactive management can be improved, thereby contributing to minimizing hospitalizations and, consequently, reducing healthcare-associated costs. This advancement in telemedicine provides a viable and efficient solution for caring for patients with respiratory conditions, simultaneously addressing challenges related to geographic distance and accessibility to pulmonary rehabilitation programs Top of Form [16,17,18,19,20].

It is an innovative medical strategy aimed at improving the quality of care and access to medical services in rural regions. However, there is a limitation in evidence regarding how telemedicine influences the relational dynamics between medical institutions and local communities. Current studies have revealed a lack of data concerning the specific impact of telemedicine on the relationship between the hospital and the community, underscoring the need for further research in this field. Evaluating this dynamic can provide essential insights for optimizing the implementation of telemedicine to support rural communities in accessing and benefiting from quality medical services [21].

The utilization of telemedicine may encounter several obstacles and risks. Legal impediments within the realm of telemedicine include the possibility that teleconsultations may fall below the established standard of care. There is also the potential for equipment or systems to experience failures. Electronic data are susceptible to manipulation, and electronic records are vulnerable to abuse. Additionally, networks used for telemedicine may have inadequate data protection, which compromises confidentiality, authenticity, data reporting, procedure certification, and overall security and privacy. Another challenge is determining the responsibilities and potential obligations of health professionals within the network [22].

In addition to these legal impediments, there are other obstacles to telemedicine. One major issue is insufficient familiarity with eHealth among patients, citizens, and healthcare professionals. There is also inadequate interoperability among diverse telemedicine solutions. The substantiation of the cost-effectiveness of telemedicine remains restricted, and legal challenges and apprehensions persist. There is an absence of transparency regarding data utilization, inadequate reimbursement frameworks, and substantial initial implementation expenses [22].

When adopting telemedicine for asthma patients, healthcare professionals must consider ten principles to guide the implementation of this system. First, they need to understand the different types of telemedicine and use the appropriate ones for treating asthma patients. It is essential to stay informed about the latest regulations regarding telemedicine at both state and federal levels. Selecting a suitable platform and establishing the necessary infrastructure are critical steps. Healthcare professionals should provide telemedicine consultations, obtain informed consent, and make necessary preparations ahead of each visit. During the consultation, they must attend to the patient and conduct a physical examination if needed. Finally, they should generate a bill for the telemedicine appointment [13].

The objective of this research was to assess the impact of utilizing home monitoring devices in the treatment of asthma on clinical outcomes and its feasibility for long-term use. In the specific context of asthma management, this article aims to investigate the efficacy and acceptance of home spirometry and telemedicine as methods for improving self-monitoring and patient engagement.

## 2. Materials and Methods

The selection of articles for this literature review adhered to the guidelines of PRISMA (Preferred Reporting Items for Systematic Reviews and Meta-Analyses). The literature search was carried out in May 2024 utilizing the PubMed database, Web of Science, Scopus, and Cochrane Library, with a focus on studies published from 2014 to 2024, encompassing a span of 10 years. We utilized the following MeSH terms for the PubMed search: ((“telemedicine” [MeSH Terms]) AND (“severe asthma” [MeSH Terms])) AND ((“asthma” [MeSH Terms]) AND (“home spirometry” [MeSH Terms])), (tele-pulmonary rehabilitation [MeSH Terms]) AND (asthma [MeSH Terms]). The search on Web of Science was conducted using the topic option that focuses on analysis. Queries are conducted on the title, abstract, keywords plus, and author keyword fields, utilizing the specified keywords as follows: “asthma control”, “home monitoring systems”, “home spirometry”, ”tele-pulmonary rehabilitation”, “portable spirometers”, and “asthma telemedicine”. The same protocol was used for Scopus and Cochrane Library searches. The search was restricted to journal articles written in English. A methodical and systematic search strategy was employed to identify pertinent scientific papers that investigate the utilization and efficacy of telemedicine in patients with asthma, according to the PRISMA statement and checklist [23].

The pre-established set of exclusion criteria comprised the following:

(1) Studies not in the English language;
(2) Articles published over 10 years ago;
(3) Research not involving human subjects;
(4) Studies conducted on individuals under 18 years old;
(5) Research not utilizing telemedicine as an alternative follow-up method;
(6) Studies focusing on pathologies other than asthma;
(7) Reviews about telemedicine and asthma.

**Figure 1:**
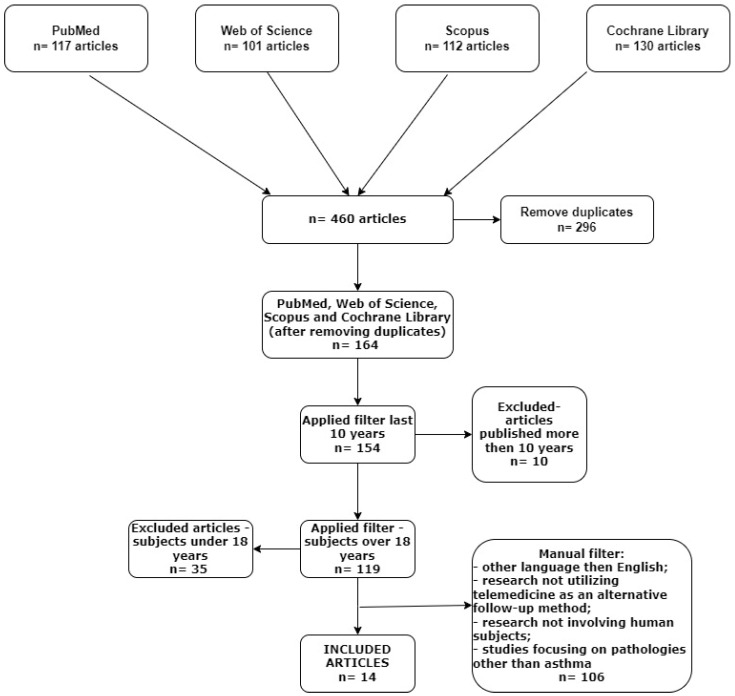
PRISMA flowchart. Literature review.

The diagram below illustrates the employed search strategy and the implemented filters. A step-by-step algorithm based on the PRISMA statement is presented in Figure 1.

## 3. Results

In the course of this systematic review, a thorough analysis was conducted on 14 articles, encompassing a total of 9093 patients. Table 1 presents the attributes of the analyzed studies.

### 3.1. Home Spirometry

Often, asthmatic patients delay seeing a doctor because they feel better and only come when they need medication. We conducted an analysis of several studies on the use of mobile spirometers in adult patients. Although most research has focused on children, there are also relevant studies for the adult population.

According to the single-arm, open-cohort, multicenter trial by Kupczyk M. et al. (2020), which evaluated the viability and security of a portable respirometry device, the following results were found: A total of 67 individuals (86%) out of 78 with full data met the main objective. During the three-week follow-up, 75 participants (96%) used the gadget correctly at least once, and 10 patients (13%) achieved success each and every day. Three patients’ data were missing because of wi-fi connection issues, while five patients revoked their consent. There was a difference in the satisfactory respirometry examination rate (p 0.013) between the sites. Of the 62 eligible patients, 20 (32%) required retraining, and 8 (40%) were successful. Contentment with the AioCareVR system scored highly; 90% of respondents said it was practical and easy to use [31].

In the study conducted by Potonos D. et al., adults with difficult-to-manage or severe asthma were sequentially instructed on how to use the AirNext spirometer (NuvoAir US Inc., Boston, USA)and were tasked with conducting spirometry at home on a daily basis for the initial month, followed by weekly sessions or when symptoms worsened. Geolocation data were utilized to distinguish between patients’ initial spirometry sessions conducted in the hospital (referred to as ‘supervised’) and those conducted at home (referred to as ‘unsupervised’). Informed consent was obtained from all patients. A total of ten asthma patients were included, consisting of four individuals with uncontrolled asthma and six with partially controlled asthma, as per the GINA 2018 guidelines. Among these patients, 90% were female, with an average age of 42.0 ± 10.7 years, and an FEV1 of 69.6 ± 26.2% (ranging from 27.3 to 101.0) predicted during the supervised session. Throughout the study period, these patients collectively performed 1235 unsupervised sessions spanning an average duration of 181.5 ± 153.9 days (with a maximum of 382 days). Of these sessions, 877 (71.0%) met the criteria for acceptable quality (graded as A–C, involving at least two maneuvers with <200 mL FEV1 and FVC variation), with the majority (556 sessions) graded as A. In contrast, only 50.0% of the 10 supervised sessions were considered acceptable. The assessment of spirometry session quality was determined according to the guidelines outlined in the American Thoracic Society Technical Statement. When the spirometry results were analyzed, it was observed that the supervised tests conducted in the hospital yielded outcomes similar to the initial unsupervised tests performed at home. Specifically, the FEV1 and FVC measurements were 2.1 ± 0.3 L and 2.9 ± 0.3 L, respectively, during the supervised evaluations, compared to 2.2 ± 0.2 L and 2.9 ± 0.2 L, respectively, during the initial unsupervised home evaluations. Additionally, the average predicted FEV1 values were 69.6 ± 26.2% and 72.0 ± 24.0%, respectively [36].

Bindler et al. conducted a study to assess if regular monitoring of young adults with asthma improved airway function, measured by FEV1, and to evaluate the feasibility of self-administered spirometry. In total, 67 participants (ages 18–26) used a portable spirometer connected to a smartphone app, with data collected at baseline, week 4, and week 8. Compliance was high, with 100% completing spirometry at baseline, decreasing to 94% at week 4 and 86.6% at week 8. FEV1 levels remained stable throughout the study, and over two-thirds of participants used the app to document their symptoms and triggers. The study found that self-administered spirometry is a feasible and acceptable method for young adults [35].

### 3.2. Clinic Versus Home Spirometry

We also found some studies comparing spirometry performed at home with those conducted in the hospital.

In this study, Huang C. et al. wanted to see if mobile spirometry could provide more statistical power than its clinical equivalents. For twice-daily and at least once-daily use of mSpirometry, the mean subject compliance was 70% and 85%, respectively. The FEV1 readings from mSpirometry showed a strong correlation and agreement with those from the clinic taken the same morning (r = 0.993) and afternoon (r = 0.988). The interclass correlation coefficient (ICC) was used to evaluate the test–retest reliability for mSpirometry and clinic spirometry. The mean difference between the afternoon (0.0019 L) and morning (0.0126 L) measures was less. Both mobile (ICC = 0.932) and clinic (ICC = 0.942) spirometry demonstrated similar test–retest reliability. Compared to sparse clinic measurements, this simulation study showed that dense mSpirometry had more power. They have shown higher statistical power, strong agreement, and comparable test–retest reliability with clinic equivalents for repeated at-home mSpirometry, which may have applications in asthma clinical research [32].

Oppenheimer J. et al. conducted the largest asthma study to date by comparing spirometry performed at home and in a clinic in this post hoc analysis. The randomized double-anonymized parallel-group phase 3A CAPTAIN (205715; NCT02924688) and phase 2B 205832 (NCT03012061) studies, which included patients with uncontrolled asthma, provided the trough FEV1 data used in this retrospective study. The 205832 study looked at the effects of adding umeclidinium to fluticasone furoate in comparison to a placebo, while the CAPTAIN trial studied the effects of adding umeclidinium to fluticasone furoate/vilanterol using a single inhaler. Both at-home and under-supervision in-person spirometry were used to determine trough FEV1. The measurements were taken at the study clinic. The authors created post hoc Bland–Altman plots to evaluate agreement between the two approaches and time-course assessments of FEV1 at home and in the clinic. An analysis was conducted on data from 421 patients (205832 study) and 2436 patients (CAPTAIN trial). In both studies, treatment-related increases in FEV1 were noted through the use of both clinic and home spirometry. When compared to measurements taken in a clinic, the gains determined by home spirometry were, however, less significant and less reliable. Poor agreement was shown by Bland–Altman plots between FEV1 at baseline and week 24 in the home and clinic. The findings imply that unsupervised home spirometry readings cannot be used as a straight replacement for clinic measures since they are less reliable and agree less with the results of clinic spirometry. These results, however, might only apply to home spirometry with the specific equipment and coaching techniques used in these investigations. To make the best use of home spirometry after the pandemic, more studies are necessary [38].

### 3.3. Self-Management

It is particularly important for the patient to be aware of their illness, understand it, and manage it independently.

Guarnieri et al. created a custom questionnaire that was emailed to 180 patients with severe asthma. The majority of these patients, 82%, were comfortable with the concept of self-monitoring and managing their condition. Additionally, 77% of the participants were in favor of conducting virtual consultations and utilizing telemedicine. When it came to home treatment, 93% found self-injection therapy to be straightforward, 94% felt secure, and 93% had no concerns about administering the treatment themselves. Only a small percentage, 22%, reported minor side effects following self-administration. The study showed a consensus between healthcare professionals and patients on the feasibility of disease treatment and monitoring via telehealth, with biologics proving to be safe and easily self-administered at home [33].

This observational study, coordinated by Kuipers et al., involved 21 community pharmacies in the Netherlands. It aimed to evaluate the accuracy of electronic inhalation monitoring device (EIMD) registrations by comparing them with patient-reported inhalations. A total of 32 patients from 18 pharmacies participated, confirming 932 medication doses. The study found a 3.5% false-positive rate (inaccurate device registrations), an 85.4% true-positive rate (accurate registrations), and a positive predictive value of 96.0%. While some patients were initially hesitant about using the EIMD, most found it acceptable and easy to use, though they desired more personalized features in the app. Improving accuracy and user-tailored features were highlighted for enhancing device usability and acceptability [28].

### 3.4. Remote Digital Coaching—Follow Up

In a 12-week remote digital coaching program by Rasulnia et al., 51 adults with uncontrolled asthma were enrolled. The program used various tools and strategies to encourage self-management of the disease. Out of the initial participants, 40 completed the study. Significant improvements were observed in mental status, body weight, and frequency of outpatient exacerbations. Changes in Asthma Control Test scores were statistically significant but did not meet the minimum clinically important difference. However, the Asthma Symptom Utility Index scores achieved both statistical significance and clinical importance. No changes were noted in lung function and rescue albuterol usage [25].

Prabhakaran et al. conducted a multicenter randomized controlled trial in Singapore involving emergency room patients diagnosed with asthma from March 2013 to February 2015. Patients were randomly assigned to either the eCARE intervention group (*n* = 212) receiving SMS messages or a control group (*n* = 212) receiving regular care. After five weeks, there was no significant difference in achieving well-controlled asthma between the groups initially with poorly controlled asthma. Logistic regression at three months suggested a trend towards better asthma control in the usual care group, but this was not statistically significant. Despite high satisfaction (95%) with the SMS service among eCARE participants, the intervention did not lead to improved asthma control compared to standard care [30].

## 4. Complementary Telemedicine Tools

The use of technology by some patients can be problematic. They need a smart device with Bluetooth Smart connectivity and internet access to download the application and regularly send spirometry results to the specialist.

The progress in technology, particularly in personal health monitoring, is truly remarkable. Devices like smartwatches and other gadgets are now equipped with specialized sensors capable of tracking numerous physiological parameters. This gives users an in-depth understanding of their health. These devices can record heart rate, monitor sleep quality, and track physical activity, becoming more advanced and helpful in preserving and enhancing our health. However, it is crucial to be mindful of data privacy and understand how these data are stored and utilized by device manufacturers.

There are various types of lung monitoring devices designed for home use, to help track lung function and monitor aspects of respiratory conditions such as asthma and COPD. Here are some examples as follows:

**Mobile spirometer:** This serves as a diagnostic and monitoring tool for lung conditions. It allows us to measure the patient’s lung volumes to see how well the condition is being managed [39].

**Peak flow meter:** This is a quick test that measures the peak expiratory flow rate and is beneficial for self-monitoring at home, both for managing the disease and assessing how well the patient responds to treatment [39].

**Pulse oximeter**: This small device attaches to a person’s fingertip to measure oxygen saturation. It is frequently used to monitor individuals with respiratory disorders [39].

**Portable capnograph:** This measures the concentration of carbon dioxide in exhaled breath. Monitoring the end-tidal carbon dioxide is crucial in observing patients on mechanical ventilation to guarantee sufficient ventilation. Although primarily used in hospitals to monitor breathing during anesthesia or intensive care, portable versions are available for home use [40].

**Smart inhalers:** These electronic monitoring devices connect to a smartphone app to track medication usage, inhalation techniques, and other relevant data. They help individuals with asthma or COPD manage their treatment, monitor symptoms, and receive reminders for medication administration. In the current technological landscape, it has become viable to integrate the geographical data of inhaler usage by patients with environmental metrics. This integration facilitates the identification of regions that exhibit a higher susceptibility to asthma attacks. Several studies have established that the implementation of reminders via electronic inhalers significantly bolsters adherence to medication regimens, presenting a marked improvement over traditional therapeutic approaches [41,42,43,44].

**ADAMM (Automated Device for Asthma Monitoring and Management):** This innovative lung monitoring device uses artificial intelligence (AI) to help individuals with asthma efficiently manage their symptoms. ADAMM is a wearable device that monitors breathing patterns, heart rate, and physical activity. Equipped with sensors that detect changes in breathing, such as snoring or shortness of breath, it uses AI to analyze the data and provide personalized feedback and recommendations for managing asthma symptoms [39,45,46].

**Portable FeNo:** This method is quantitative, noninvasive, straightforward, and safe for gauging inflammation in the airways. It serves as an additional resource to other techniques used for evaluating diseases of the airways and it is recommended for diagnosing eosinophilic airway inflammation [47,48,49].

## 5. Ensuring Privacy and Data Protection in Telemedicine

There are serious privacy problems associated with telemedicine because it employs electronic medical records (EMRs) to record follow-up information, diagnoses, medications, and medical histories. The protection of patient records is variable due to variations in federal laws. Patient autonomy, safety, cultural diversity, and informed consent are examples of ethical dilemmas. Studies emphasize the need for informed consent while pointing out the inconsistencies in its implementation. It is advised that data protection be governed by uniform regulations such as the General Data Protection Regulation (GDPR), with a particular focus on protecting the data of children and families. Although there are always malpractice worries, telemedicine is generally more advantageous than harmful. There are proposals for uniform legislation due to the fragmentation of legal regulations. The benefits of telemedicine in terms of reduced costs and better care are widely recognized, yet cultural sensitivity is still very important. The potential for AI integration to expand the reach and effectiveness of telemedicine is anticipated [50].

**Informed consent:** Confidentiality is essential in telemedicine. Providers need to obtain explicit consent and provide an explanation for data access and use. Identity validation, perhaps with the use of photo IDs, must come first in sessions. In addition to ensuring secure data transmission and storage, providers also need to educate patients on the value of secure networks. Emergency care is covered by these guidelines as well [51].

Protection of data and confidentiality: The world’s harshest privacy and security law was enacted on 25 May 2018, and it is called the GDPR. Despite having been developed by the European Union (EU), it is applicable to any entity globally that targets or gathers personal data about individuals within the EU. According to the GDPR’s precise definition of data points, data protection for medical information has improved. Adopting comparable consistent guidelines globally is advised by experts to guarantee optimal confidentiality and protection of data [52,53].

Laws and regulations: The necessity for unified regulation is shown by the disjointed approach to telehealth laws. European studies deal with the application and harmonization of directives, while North American studies concentrate on interstate laws and licensing. Entire and uniform regulations are necessary for telehealth to function well [54].

## 6. Discussion

***In the instance of telemedicine, which approach appears to be promising for asthmatic patients?*** Mobile spirometers together with eHealth inhalers can become promising methods for monitoring in telemedicine, provided that patients are properly trained in their use at home. Ensuring proper training is crucial to maximize their benefits.

Most studies on remote spirometry and telemedicine in asthma often focus on children and adolescents. These studies explore various aspects such as the effectiveness of remote monitoring, adherence to the treatment, symptom management, and the impact on quality of life. 

We live in a modern era where technology is advancing rapidly in all fields, astonishing us with innovations we could not have imagined just a few years ago. There have been remote spirometry techniques for a while, and as smartphones and tablets become more popular, there is a greater need for portable medical devices like spirometers that can pair with apps using Bluetooth [55,56].

According to the research by Finkelstein and Jeong, machine learning techniques have a lot of potential for creating customized decision assistance for telemonitoring systems that target chronic illnesses. The 7001 daily self-monitoring reports from adult asthma patients undertaking home telemonitoring made up the study dataset. Choosing predictive characteristics, building stratified training datasets, and evaluating the resulting classifiers were all part of predictive modeling. Asthma exacerbations on the eighth day were successfully predicted by using a 7-day window, a naive Bayesian classifier, an adaptive Bayesian network, and support vector machines. Sensitivity values of 0.80, 1.00, and 0.84; specificity values of 0.77, 1.00, and 0.80; and accuracy rates of 0.77, 1.00, and 0.80, respectively, were attained [57].

In recent years, several devices for monitoring asthma have been developed, and the study conducted by Morita et al. presents one of these applications. Breathe is a web-based mobile health (mHealth) platform that can be accessed on desktop, tablet, or smartphone devices. It was created using quality development standards approved by the International Organization for Standardization and user-centered design techniques. Breathe was also intended to be a multipurpose collaborative self-management (CSM) mHealth platform with material derived from compliance with national privacy and security regulations and according to international clinical practice recommendations. Through (1) the evaluation of asthma control, (2) real-time access to a dynamic asthma action plan, (3) access to real-time environmental circumstances, and (4) risk-reduction messaging, the system enabled CSM (patient, provider, and breathe) and self-monitoring of asthma patients. The data-collecting procedure involved baseline clinic visits as well as follow-up visits every six and twelve months to gather user data. User perceptions, self-reported medicine usage, the profile of asthma symptoms, reported peak flow measurements, and the effectiveness and delivery of email reminders were among the use outcomes [58].

In order to develop the intervention, Mammen et al. focused on stakeholder demands and system capacities using May’s implementation theory and a contextually grounded intervention creation technique. The intervention included smartphone-based telemedicine sessions, self-management instruction with a nurse, and symptom monitoring. Clinical decision-support software that offered step-by-step therapy, automated estimations of asthma severity, and control was used. A three-month beta test was conducted with seven people (ages 18 to 40). At baseline and study completion, asthma outcomes (control, quality of life, and FEV1) and patterns of healthcare utilization were assessed [59].

Ghulam Nabi et al. developed a telemedicine application using machine learning to automatically classify the severity of asthma. They used respiratory sounds from 111 patients and classified them into mild, moderate, and severe categories. The application, based on Mel-frequency cepstral coefficient (MFCC) features, can detect wheezing and classify its severity. The classification accuracy was highest for mild cases at 99%, with an average wheeze detection rate of 93%. The validation accuracy of the application was highest for severe levels at 76% [60]. While some studies have focused on patient monitoring, there are also studies that address the quality of life of asthmatic patients. Harada et al. used ResearchKit (ResearchKit platform of Medical Logue, Inc. (The Zensoku-Log)) to develop an app and conducted a survey on patients with cough variant asthma and asthma via mobile devices from February 2016 to February 2018. The app was downloaded 7855 times and gathered data on symptoms, comorbidities, medications, asthma control, and adherence from 1744 participants (median age 33 years; range 20–74 years; male-to-female ratio 38.7:61.3). The results showed high rates of pet ownership (40.75%), smoking (23.14%), and unplanned healthcare visits (50.97%). Most participants had atopic predispositions (91.89%) and received daily inhaled corticosteroids (89.45%) or oral corticosteroids (22.07%). Despite this, many had poor asthma control, with limited use of additional treatments like leukotriene receptor antagonists (30.66%), theophylline (15.91%), and long-acting muscarinic antagonists (4.38%). Regular app use correlated with improved asthma management, highlighting potential gaps in treatment for Japanese mHealth app users [61]. According to Himes et al., asthma management can benefit from wearables, web apps, and mobile health technologies, with around 500 asthma-related apps available for education, symptom tracking, and medication reminders. These tools collect data on symptoms and inhaler use, helping detect worsening conditions and combining with environmental data to predict triggers. Challenges include privacy concerns, cost, user engagement, and ensuring accessibility for diverse populations. Despite these issues, research shows that these technologies have significant potential to improve asthma care and patient–doctor interactions [62]. There have been studies investigating asthmatic patients’ perceptions regarding telemedicine. Studies of this kind are important for understanding how telemedicine can be integrated into medical practice to improve access to care and the quality of life for asthma patients. One of these studies was conducted by Sousa and his collaborators. The study involved a questionnaire posted on the Portuguese Association of Asthmatics’ Facebook page, starting on 11 May 2020, available for self-reported asthmatic patients to complete online over a month. Out of 55 responses received, over 88% of patients were satisfied with their communication with doctors, yet 50% found virtual visits less effective than in-person ones. Despite some patients rating virtual visits lower on a scale from 0 to 10, most indicated they would still use or recommend them in the future, beyond the pandemic. Drawbacks included the absence of physical examinations and a perceived impersonal nature, though only 27% faced technological difficulties. Overall, while adjustments are needed, the study suggests virtual visits are generally well received and could serve as a viable alternative to in-person visits, particularly during times of social distancing [63].

The studies analyzed in this systematic review share the common use of telemedicine through various methods, including mobile spirometers and mobile phone applications. These approaches aim to improve communication, information, education, adherence control, and the quality of life of patients. Although we do not present groundbreaking innovations in the medical field, we have succeeded in centralizing all the essential information needed by both patients and healthcare professionals to understand and effectively implement telemedicine.

## 7. Limitations and Future Directions

Besides the limitations of a systematic review, the actual study demonstrates that home monitoring systems, including mobile spirometry and telemedicine, can positively impact asthma management. While challenges remain, technological advancements and AI integration hold promise for improving clinical outcomes and reducing healthcare costs. Future research should focus on optimizing these technologies to enhance their reliability and user acceptance.

## 8. Conclusions

While telemedicine was once primarily used in remote or rural areas, it has now grown and broadened to meet worldwide health demands. Elements like convenience, efficiency, communication, and privacy play a crucial role in the acceptance of telemedicine, which aids in the interaction between patients and healthcare providers. Nonetheless, there are ongoing hurdles and challenges in the deployment of telemedicine that need to be overcome, such as dealing with technological obstacles and assuring the quality and safety of medical services provided remotely. By tackling these issues, telemedicine has the potential to become a substantial asset in healthcare, offering significant advantages to both patients and healthcare professionals.

In the context of asthma management, home spirometry and telemedicine offer promising methods for improving self-monitoring and patient engagement, potentially reducing the frequency of clinic visits. Studies indicate that with proper training, home spirometry can provide reliable data comparable to clinic-based measurements. Additionally, patients are generally receptive to telemedicine and self-management tools, although further improvements in technology and training are needed to maximize their benefits and reliability. These advancements, coupled with machine learning and digital health platforms, hold significant potential for enhancing asthma care and patient quality of life.

## Figures and Tables

**Table 1:** Attributes of the analyzed studies.

Authors (Publication Year)	Study Location	No. of Patients	Age of Patients (Mean-SD)	Duration of Study	Telemedicine Tools	Outcome Category	Results
**Honkoop et al.** **(2017) [24]**	UK Netherlands	150	≥18	1 year	Portable spirometer; Portable FeNo; Device for exhale breath temperature, RR, physical activity, HR; Smartinhaler device; Pollen forecast	Asthma control; Self-management Quality of life	Positive impact of telemedicine on asthma control, self-management, and quality of life.
**Rasulnia et al. (2017) [25]**	US	51	≥18	12 weeks	E-mail; Phone call; SMS	Quality of life; Asthma control	The digital coaching program, using experienced health coaches and digital tools, significantly improved mental health, and decreased outpatient exacerbations, body weight, and ASUI.
**Chan et al. (2018) [26]**	US	7593	≥18	6 months	Mobile application	Feasibility; Asthma control	There is a rise in the number of reported asthma symptoms in areas impacted by heat, pollen, and wildfires.
**Nemanic et al. (2018) [27]**	Slovenia	100	≥18	1 year	Web-based; SMS	Asthma control; Self-monitoring	The success of a newly developed home monitoring program, utilizing technology, in aiding asthma patients manage their disease effectively.
**Kuipers et al. (2019) [28]**	Netherlands	32	≥18	5 weeks	E-inhalation; Phone call; Mobile app; Web-based	Self-management	Bluetooth connectivity and data synchronization are necessary. Tailoring features to individual needs could enhance the program’s acceptability.
**Rudin et al. (2019) [29]**	US	26	54 (16)	25 weeks	Mobile application; Web-based; Phone call	Symptom monitoring	Robust patient involvement and commitment to adhering to guidelines for monitoring asthma symptoms, while keeping the workload on clinicians to a minimum.
**Prabhakaran et al. (2019) [30]**	Singapore	424	≥18	2 years	SMS	Asthma control; Home monitoring	Telemedicine through SMS did not have a positive effect on those in the eCARE program.
**Kupczyk et al.** **(2020) [31]**	Poland	86	37.4 (11.4)	3 weeks	Portable spirometer; Mobile application	Symptom control; Self-monitoring	A positive impact of telemedicine on self-monitoring and symptom control.
**Huang et al. (2021) [32]**	UK	12	41.1 (9.9)	28 days	Mobile spirometer; Mobile application	Monitoring; Compliance	A positive impact of telemedicine on monitoring and compliance of the patient.
**Guarnieri et al. (2022) [33]**	Italy	180	≥18	Not specified	E-mail; Mobile spirometer; Oxyhemoglobin saturation meter	Self-injection therapy; Self-measurements	The importance of extending telemedicine to general patients with chronic respiratory diseases.
**Furci et al. (2022) [34]**	Italy	302	56	2 months	Portable spirometer; Web-based	Collaboration between hospitals	The importance of implementing integrated management across hospital and community settings to attain effective asthma control.
**Bindler et al. (2023) [35]**	USA	67	≥18	2 months	Portable spirometer; Mobile application	Self-measurements; Self-monitoring	Self-administered spirometry is a feasible and acceptable method for young adults.
**Potonos et al. (2023) [36]**	Greece	10	42.0 (10.7)	382 days	Portable spirometer; Mobile application	Follow up	Home spirometry performed without supervision can yield results of high quality.
**Izmailova et al. (2023) [37]**	US	60	≥18	24 weeks	Mobile spirometer; Mobile application; Wrist-worn	Treatment effect; Adherence; Accuracy; Asthma control	A positive impact of telemedicine on the early detection of exacerbations, decreased expenses linked to clinic appointments, and improved safety for patients.

SD = standard deviation; RR = respiratory rate; HR = heart rate; SMS = Short Message Service.

## Data Availability

The data are encapsulated within this article. Further details can be obtained upon request from either the primary author or the corresponding author.
